# Whole-genome analysis of human papillomavirus 67 isolated from Japanese women with cervical lesions

**DOI:** 10.1186/s12985-022-01894-z

**Published:** 2022-10-07

**Authors:** Gota Kogure, Mamiko Onuki, Yusuke Hirose, Mayuko Yamaguchi-Naka, Seiichiro Mori, Takashi Iwata, Kazunari Kondo, Akihiko Sekizawa, Koji Matsumoto, Iwao Kukimoto

**Affiliations:** 1 Pathogen Genomics Center, National Institute of Infectious Diseases, Tokyo, Japan; 2 Department of Obstetrics and Gynecology, Showa University School of Medicine, Tokyo, Japan; 3 Department of Obstetrics and Gynecology, Keio University School of Medicine, Tokyo, Japan; 4 Department of Obstetrics and Gynecology, NTT Medical Center Tokyo, Tokyo, Japan

**Keywords:** Human papillomavirus, Type 67, Variant, Long control region, Cervical cancer

## Abstract

**Background:**

Human papillomavirus (HPV) type 67 is phylogenetically classified into *Alphapapillomavirus* species 9 (alpha-9) together with other carcinogenic types (HPV16, 31, 33, 35, 52 and 58), but is the only alpha-9 type defined as possibly carcinogenic. This study aimed to determine whole-genome sequences of HPV67 isolated from Japanese women with cervical cancer or cervical intraepithelial neoplasia (CIN) to better understand the genetic basis of the oncogenic potential of HPV67.

**Methods:**

Total cellular DNA isolated from cervical exfoliated cells that were single positive for HPV67 (invasive cervical cancer, n = 2; CIN3, n = 6; CIN 2, n = 1; CIN1, n = 2; the normal cervix, n = 1) was subjected to PCR to amplify HPV67 DNA, followed by next generation sequencing to determine the complete viral genome sequences. Variant lineages/sublineages were assigned through viral whole-genome phylogenetic analysis. The transcriptional activity of the virus early promoter was assessed by luciferase reporter assays in cervical cancer cell lines.

**Results:**

Phylogenetic analyses of HPV67 genomes from Japan (n = 12) revealed that 11 belonged to lineage A (sublineage A1, n = 9; sublineage A2, n = 2) and one belonged to lineage B. All cancer cases contained sublineage A1 variants, and one of these contained an in-frame deletion in the *E2* gene. The long control region of the HPV67 genome did not induce transcription from the virus early promoter in HeLa cells, but showed weak transcriptional activity in CaSki cells.

**Conclusions:**

The single detection of HPV67 in cervical cancer and precancer specimens strongly suggests the carcinogenic potential of this rare genotype. The phylogenetic analysis indicates a predominance of lineage A variants among HPV67 infections in Japan. Since only 23 complete genome sequences of HPV67 had been obtained until now, the newly determined genome sequences in this study are expected to contribute to further functional and evolutionary studies on the genetic diversity of HPV67.

**Supplementary Information:**

The online version contains supplementary material available at 10.1186/s12985-022-01894-z.

## Background

Approximately 13 genotypes of human papillomavirus (HPV) are responsible for the development of cervical cancer [1]. Among them, HPV16 is the most frequently detected type in invasive cervical cancer (ICC), followed by HPV18, and these two types account for about 70% of ICC cases worldwide [2]. The degree of carcinogenicity of each HPV type has been determined on the basis of epidemiological data showing that each type is detected as a single infection in ICC. Accordingly, the International Agency for Research on Cancer classified HPV16, 18, 31, 33, 35, 39, 45, 51, 52, 56, 58 and 59 as carcinogenic (Group 1), HPV68 as probably carcinogenic (Group 2A), and HPV26, 53, 66, 67, 70, 73 and 82 as possibly carcinogenic (Group 2B) [3].

HPV67 was originally isolated from a Japanese woman with vaginal intraepithelial neoplasia [4], but it is rarely found in cervical cancer. Despite its low prevalence, HPV67 is reliably detected as a single infection in a small percentage of ICC cases, suggesting its carcinogenicity [5]. According to a worldwide meta-analysis, the prevalence of HPV67 in ICC was estimated to be 0.3%, the twentieth most frequent type in ICC [6]. However, the high single/multiple infection ratio of HPV67 in ICC cases, as well as that of Group 1 types, supports its causative role in cervical cancer development [7]. In a recent study that analyzed HPV single type infections in Japan, 1.0% (3/306) of ICC cases were attributed to HPV67 infections, and these HPV67 single-positive cases were all squamous cell carcinoma (SCC) [8].

Based on the viral whole-genome sequence, HPV67 is phylogenetically classified into *Alphapapillomavirus* species 9 (alpha-9) together with other highly carcinogenic types, HPV16, 31, 33, 35, 52 and 58 [9]. Interestingly, HPV67 is the only alpha-9 type not currently categorized as Group 1 but is defined as Group 2B. However, the reason for the different prevalence in ICC between HPV67 and the other alpha-9 types is still unknown.

In the present study, we sought to determine whole-genome sequences of HPV67 isolated from Japanese women with cervical lesions, and analyzed viral variant lineages/sublineages and genetic variations. Moreover, the transcriptional activity of the virus long control region (LCR) was compared between HPV67 and other carcinogenic types using cervical cancer cell lines.

## Methods

### Clinical specimens

Cervical exfoliated cells were collected using a cytobrush from Japanese women who visited the Department of Gynecology at Keio University Hospital, NTT Medical Center Tokyo, and Showa University Hospital, as outpatients having some sort of symptoms or referred by primary care physicians for further examination, from 2009 to 2018. Total cellular DNA was extracted from the collected cells using the MagNA Pure LC Total Nucleic Acid Isolation kit (Roche, Indianapolis, IN) on a MagNA Pure LC 2.0 (Roche), and subjected to HPV genotyping by PGMY PCR followed by reverse line blot hybridization as described previously [10].

### Viral whole-genome sequencing

Two overlapping PCRs that cover the whole-genome sequence of HPV67 were performed with HPV67-positive DNA samples using PrimeSTAR® GXL DNA polymerase (Takara, Ohtsu, Japan) or KOD One® PCR Master Mix (Toyobo, Osaka, Japan) with the following primers: HPV67-631F (5′-CTG CTA TGA GCA ATT GCA TGA CAG CTC-3′) and HPV67-4627R (5′-CCA GAT GCT GTT GGA ATA GAT GGT GCA-3′); HPV67-4261F (5′-CTA CAA GGC GCA AAC GTG CCT CTG CAA CAC-3′) and HPV67-1100R (5′-CAC TAC CAG TGT CAG ATG CAT CCT CAT CCT-3′). The PCR protocol included 38 cycles of 98 °C for 15 s, 55 °C for 30 s, and 68 °C for 2 min. The amplified DNA, separated on an agarose gel and purified with the Wizard gel purification kit (Promega, Madison, WI), was converted to a short-fragmented DNA library using the Nextera XT DNA sample prep kit (Illumina, San Diego, CA), followed by size selection with SPRIselect (Beckman Coulter, Brea, CA). The multiplexed libraries were then sequenced on a MiSeq (Illumina) or an iSeq100 (Illmina). The VirusTAP pipeline [11] was used to de novo assemble HPV67 complete genome sequences from the short read sequences. The mean sequencing depth for samples ranged from 10,551 to 59,852. The accuracy of the reconstructed whole-genome sequences was verified by visual inspection using Integrative Genomics Viewer v2.3.90.

### Phylogenetic analysis

The HPV67 genome sequences newly determined in this study and those from GenBank (LR862038, LR861998, LR861975, LR861967, LR861960, LR861950, LR861934, LR861913, LR861905, LR861856, LR861847, LR861843, KX514425, KU298929, KU298930, HQ537784, HQ537783, HQ537782, HQ537781, HQ537780, HQ537779, HQ537778 and D21208) were aligned with each other using MAFFT v7.309. A maximum likelihood tree was constructed using RAxML HPC v8.2.9 under the general time-reversible nucleotide model with gamma-distributed rate heterogeneity and invariant sites (GTRGAMMAI) employing 1000 bootstrap replicates. The constructed phylogenetic tree was visualized in FigTree v1.4.3.

### Luciferase reporter assay

The LCR sequences of HPV67, 31, 52 and 58, were amplified from corresponding clinical samples by PCR with KODplus DNA polymerase (Toyobo) and the following primers: HPV67A1-F (5′-CCC AAG CTT TTG TAT GAC TGT TGT GTG TTT GTA-3′) and HPV67A1-R (5′-CAT GCC ATG GAT TTC TTG CAC TGT AGG TGG ACA C-3′); HPV67B-F (5′-CCC AAG CTT TTG TAT GAC TGT TTT GTG TTT GTA-3′) and HPV67B-R (5′-CAT GCC ATG GAT TTC TTG CAC TGT AGG TGG ACA C-3′); HPV31-F (5′-CCC AAG CTT TGG ATG TGT ATG TAA TAC ATG TGT-3′) and HPV31-R (5′-CAT GCC ATG GCG TCT GTA GGT TTG CAC AAA AT-3′); HPV52-F (5′-CCC AAG CTT CCA TTG TCT GTT GGG TAA TTG-3′) and HPV52-R (5′-CAT GCC ATG GCC GTG CGT TAG CTA CAC TGT G-3′); HPV58-F (5′-CCC AAG CTT TTG TTG TGG TAC TTA CAC TAT TT-3′) and HPV58-R (5′-CAT GCC ATG GTC CTG CAG TAG CCT ACC AAA A-3′) (*Hind*III and *Nco*I sites are underlined). The PCR products were digested with *Hind*III and *Nco*I, and inserted between the *Hind*III/*Nco*I sites in pGL3-Basic (Promega), a promoter-less reporter plasmid. The reporter plasmids containing the LCR of HPV16 [12] and HPV18 [13] were previously described. The LCR sequences of HPV16, 18, 31, 52 and 58 were all from sublineage A1.

HeLa cells were seeded in 24-well plates at a density of 16,000 cells/well and grown for 24 h, followed by transfection of 200 ng of the LCR reporter plasmids or pGL3-Basic together with 5 ng of pGL4.75 using the FuGENE6 reagent (Promega). CaSki cells were seeded in 24-well plates at a density of 50,000 cells/well and grown for 6 h, followed by transfection of 500 ng of the LCR reporter plasmids or pGL3-Basic together with 5 ng of pGL4.75 using the X-tremeGENE HP reagent (Roche). At 48 h after transfection, *Firefly* and *Rennilla* luciferase activities were measured with the Dual-Glo luciferase assay system (Promega) on an ARVO MX luminescence counter (PerkinElmer, Waltham, MA), and the level of transcription was quantified as the ratio of the two luciferase activities.

## Results

Of 7169 genotyped samples, 12 (0.2%) were found to be single positive for HPV67 by sequencing analysis of hybridization-negative PCR products. The histological diagnoses of these samples were as follows: negative for intraepithelial lesion or malignancy (NILM), n = 1; CIN1, n = 2; CIN2/3, n = 7; SCC, n = 2. The mean and standard deviation of the patients’ age was 43 ± 12 years. These HPV67-positive DNA samples were subjected to overlapping PCR covering the entire HPV67 genome, followed by next generation sequencing (NGS). Viral whole-genome sequences were then de novo reconstructed from short read sequences, resulting in determination of 12 complete genome sequences of HPV67. The lengths of the reconstructed genomes ranged from 7729 to 7819 bp (Table 1), and all genomes retained typical HPV genomic organization consisting of the *E6*, *E7*, *E1*, *E2*, *E4*, *E5*, *L2* and *L1* genes, the LCR and a non-coding region between *E5* and *L2*. Accession numbers for these HPV67 genomes are shown in Table 1.

**Table 1:** HPV67 single-positive specimens analyzed in this study

ID	Histology	Age	Variant	Length (bp)	Variation	Amino acid change	Accession number
#01	Normal	47	A1	7819*	T2219C	–	LC626352
#02	CIN1	27	A1	7819*	G6146A	L1 (S206N)	LC626346
#03	CIN1	43	A2	7803	G4731A	L2 (E165K)	LC664140
#04	CIN2	37	B	7806	C734T	–	LC664139
					C3977T	–	
					G5553A	L2 (D438N)	
					A5796G	–	
					G6081A	–	
					G6720A	–	
#05	CIN3	37	A1	7819*	A987C	E1 (E38A)	LC626350
#06	CIN3	37	A1	7819*	C1201T	–	LC626351
					A4757G	–	
#07	CIN3	31	A1	7819*	T1088G	E1 (L72V)	LC626353
					C3525T	E2 (L269F)	
#08	CIN3	41	A1	7819*	–	–	LC626354
#09	CIN3	40	A1	7803	A31C	–	LC664138
					T45G	–	
					G935A	E1 (E21K)	
					A1570G	–	
					G5684A	L1 (S52N)	
#10	CIN3	51	A2	7803	T1012C	–	LC626349
#11	SCC (IA1)	79	A1	7803	–	–	LC626348
#12	SCC (IIIB)	49	A1	7729*^,^**	C1056T	E1 (A61V)	LC626347
					G5223A	L2 (D359N)	
					G5908C	L1 (D157H)	

CIN: Cervical intraepithelial neoplasia, SCC: Squamous cell carcinoma

*16-bp duplication in LCR; **90-bp deletion in *E2/E4*

**Fig. 1:**
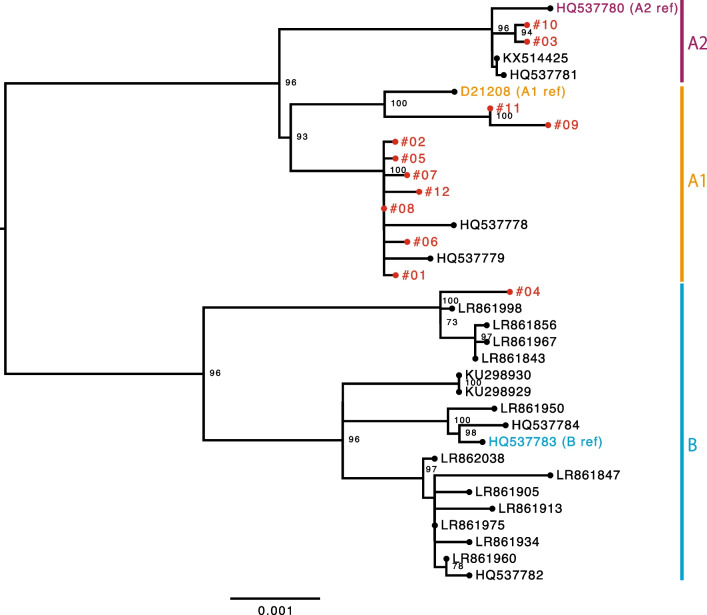
Maximum likelihood phylogenetic tree of HPV67 complete genome sequences. HPV67 genomes determined in this study are shown in red. Bootstrap values larger than 70% are shown. Scale bar, nucleotide substitutions per site

**Fig. 2:**
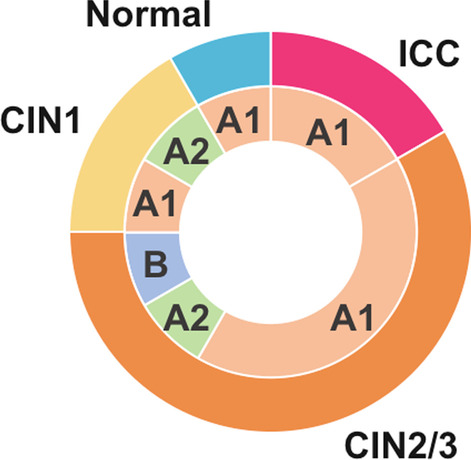
Distribution of HPV67 lineages/sublineages according to histology

As shown in Fig. 1, phylogenetic analyses of the HPV67 genomes from Japan, together with the reference genomes of HPV67 lineages/sublineages, revealed the variant assignments as follows: sublineage A1, n = 9 (75.0%); sublineage A2, n = 2 (16.7%); and lineage B, n = 1 (8.3%). These results indicate that sublineage A1 variants are dominant among HPV67 infections in Japan. The relationship between histology and variant lineages/sublineages is shown in Fig. 2 and Table 1. Two invasive cervical cancer cases contained A1 variants, while in the CIN cases, six A1, two A2 and one B variants were detected. One normal sample had an A1 genome.

Multiple sequence alignment of full-length HPV67 genomes is presented in Additional file 1: Table S1. Among the sample-specific nucleotide variations, particular non-synonymous nucleotide substitutions were found in the HPV67 genomes from Japan, leading to previously undescribed amino-acid changes in the viral proteins as follows: E1 (E21K), E1 (E38A), E1 (A61V), E1 (L72V), E2 (L269F), L1 (S52N), L1 (D157H), L1 (S206N), L2 (E165K), L2 (D359N) and L2 (D438N) (Table 1). Moreover, one cancer case (#12: SCC, stage IIIB) harbored an in-frame deletion of 90 bp in *E2*/*E4*, generating internally deleted E2/E4 proteins.

**Fig. 3:**
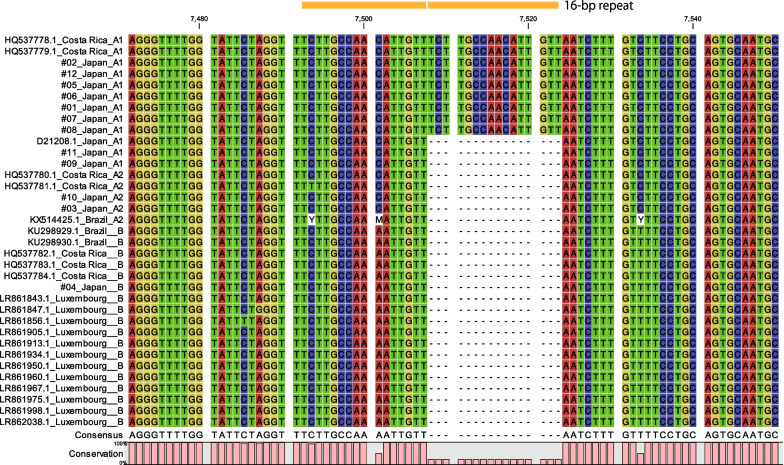
Multiple sequence alignment of the long control region of HPV67 genomes. The position of 16-bp repeats is indicated above

As shown in Fig. 3, multiple alignments of available LCR sequences of HPV67 revealed a characteristic 16-bp duplication in seven A1 genomes from Japan (#01, #02, #05, #06, #07, #08 and #12) and two A1 genomes from Costa Rica, but not in any of A2 and B genomes, two A1 genomes from Japan (#09 and #11), and D21208 (A1 reference genome). The A1 genomes with the duplicated LCR sequences were clustered together in the phylogenetic tree (Fig. 1). The JASPAR database search (http://jaspar.genereg.net/) predicted potential binding motifs for cellular transcription factors, NFI, FOXA1 and GATA3, in the duplicated sequence.

**Fig. 4:**
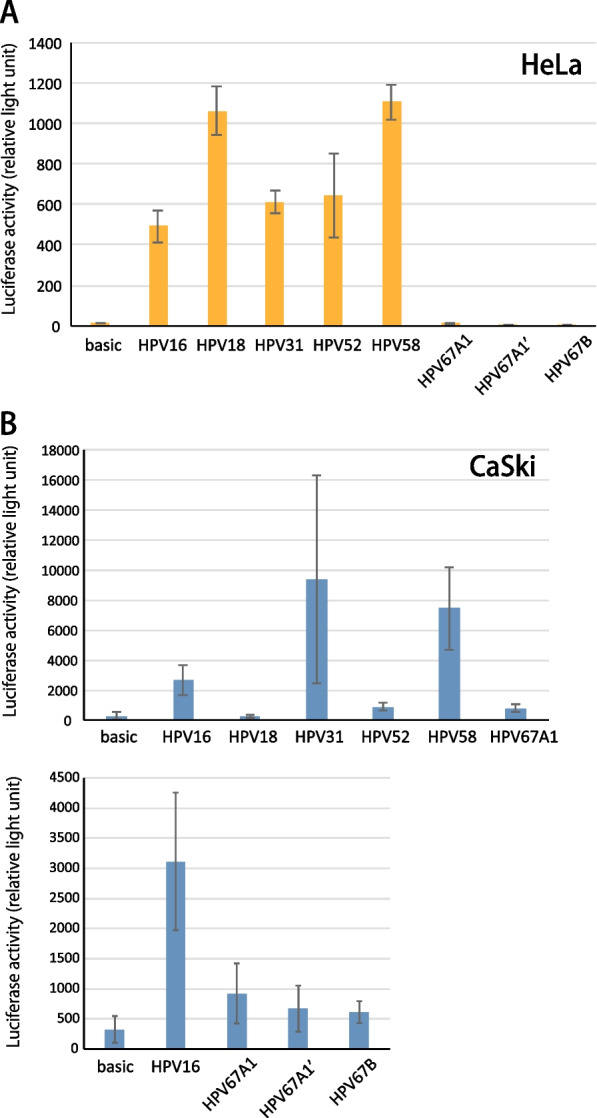
Viral early promoter activity induced by LCR of HPV16, 18, 31, 52, 58 and 67 in HeLa (**A**) and CaSki (**B**) cells. The results of reporter assays are shown as the average of three independent experiments with error bar of standard deviation. HPV67A1 and HPV67A1’ indicate LCR with or without 16-bp repeats

Luciferase reporter assays were used to investigate the transcriptional activity of the LCR of the HPV67 genome. HPV67 LCR from sublineage A1, with or without the 16-bp duplication (#12 and #11), and that from lineage B (#04) were cloned into a luciferase reporter plasmid, and examined for their transcriptional activity in HeLa and CaSki cells, in comparison with LCR from other carcinogenic types. Although LCR of HPV16, 18, 31, 52 and 58 demonstrated considerable promoter activity, none of the HPV67 LCRs induced detectable luciferase activity in HeLa cells (Fig. 4A). In contrast, HPV67 LCR showed weak but detectable transcriptional activity in another cervical cancer cell line, CaSki cells (Fig. 4B, upper panel). As previously reported [14], HPV18 LCR did not drive the viral early promoter in CaSki cells. Lastly, the sequence variations in HPV67 LCR did not significantly affect the viral promoter activity (Fig. 4B, lower panel).

## Discussion

This study is the first one to report genetic variations in the whole-genome sequence of HPV67 isolated from Japanese women. We have newly determined 12 complete genome sequences of HPV67 and performed comparative phylogenetic analyses of these HPV67 genomes with those previously reported. The results revealed that almost all of the sequenced HPV67 genomes from Japan belonged to lineage A (11/12, 91.7%), suggesting a high prevalence of HPV67 lineage A variants in Japan. The reference genome of sublineage A1 (D21208) [15] was also isolated from a Japanese woman with vaginal intraepithelial neoplasia [4]. In contrast, a recent study that used NGS for detection of multiple HPV types reported on 12 complete genome sequences of HPV67, which showed that HPV67 variant distribution in Luxembourg was totally biased toward lineage B [16], which was confirmed by our phylogenetic analysis (Fig. 1). Thus, the distributions of HPV67 variant lineages may vary geographically and ethnically, as reported for other HPV types [17, 18], likely reflecting the ancient spread of *Homo sapiens* with particular HPV variants across the world.

The presence of HPV67 single-positive ICC cases in the current study strongly supports the pathogenic role of HPV67 in generating cervical cancer. Indeed, eight probably/possibly carcinogenic types (HPV26, 53, 66, 67, 68, 70, 73 and 82) were previously shown to be biologically active in cervical cancer tissues, expressing both viral mRNA and cellular marker proteins indicative of HPV infection [19]. Furthermore, a recent study by Sakamoto et al*.* reported that out of 16 cervical cancer cases that were initially HPV-negative in liquid-based cytology samples, eight became HPV-positive by *E6/E7*-based PCR typing of microdissected archival tissue samples, among which two were HPV67 single-positive [20]. In that study, another ICC case that was initially positive for HPV6/52/55 also turned out to be single positive for HPV67. Interestingly, all three ICC cases were SCC, and this was also true for two ICC cases in our study (Table 1). Such propensity of HPV67 to SCC may reflect the transcriptional activity of its LCR observed in CaSki cells (HPV16-positive, SCC-derived cell line), but not in HeLa cells (HPV18-positive, adenocarcinoma-derived cell line). Although the LCR activity of HPV67 was much weaker than those of HPV16/31/58 in CaSki cells, the HPV67 LCR activity might be increased by cellular transcription factors differentially expressed in in vivo conditions.

Comparison of amino-acid sequences in the E6 protein between HPV67 and the other alpha-9 types revealed that HPV67 E6 has tyrosine-to-phenylalanine and glycine-to-asparagine substitutions at amino-acid positions 79 and 130 (Y79F and G130N), respectively [21]. Interestingly, these amino-acid residues are shared among non-oncogenic HPVs (species alpha-10) [21], and an HPV16 Y79N mutant was shown to be defective for degradation of p53 [22], suggesting that HPV67 E6 has a reduced biological activity compared to the other alpha-9 types, which may contribute to a low prevalence of HPV67.

The *E2* gene in the HPV genome is frequently disrupted by integration into the host genome in cervical cancer [23]. Since the E2 protein represses the HPV early promoter that drives *E6/E7* oncogene expression, inactivation or dysfunction of E2 can lead to up-regulation of *E6/E7*, thereby contributing to the development of cervical cancer. In this study, one HPV67-positive ICC case (stage IIIB) had an in-frame 90-bp deletion in the *E2* gene, which results in a 30 amino-acid deletion from position 252 to 281 of the E2 protein. Since this region corresponds to the hinge region that connects the N-terminal transactivation domain and the C-terminal DNA-binding domain [24], the deletion might affect the transcription repressor activity of E2 through changing the spatial arrangement of the two domains and facilitate cancer development.

Although epidemiological and clinical evidence is accumulating on a causal role of HPV67 in generating a minor fraction of cervical cancers, it is difficult to determine the exact prevalence of HPV67 infections in healthy women and compare that to the prevalence in women with cervical lesions. The reason for this difficulty is an inability of most HPV genotyping assays to conclusively detect HPV67. A recent systematic review that evaluated HPV genotyping assays suitable for cervical screening reported that out of 24 commercial HPV typing assays examined, only three (HPV-Risk Assay, INNO-LiPA HPV Genotyping Extra II Assay, and Linear Array HPV Genotyping Test) covered detection of HPV67, and only one of these (HPV-Risk Assay) fulfilled the validation criteria for use in cervical screening using clinician-collected specimens [25]. Other HPV genotyping assays that can detect HPV67 include Genosearch HPV31/HPV5 [8] and uniplex *E6/E7* PCR [26], both of which were used in recent studies that reported HPV67 prevalence in Japan [8, 20]. In the current study, we used PGMY PCR coupled with reverse line blot hybridization, which does not include an HPV67-specific DNA probe [10, 27]. Because HPV67 positivity was determined after sequencing hybridization-negative HPV PCR products (i.e., “HPVX” samples), multiple infections with HPV67 could not be identified, thus leading to underestimation of HPV67 prevalence in our patient cohorts.

Recently, NGS technology has been applied to detect multiple HPV types in cervical cell samples in an unbiased manner. By performing high-throughput sequencing coupled with rolling circle amplification of HPV genomes, Latsuzbaia et al*.* reported that the frequency of HPV67 infection was about 3.6% in a general population [16]. That study included 729 women in Luxembourg (aged 18 to 29) who attended regular cervical screening or visited family planning centers or gynecology practices. On the other hand, using an NGS panel that targets all carcinogenic and probably/possibly carcinogenic HPV types, Andersen et al*.* demonstrated that 5 out of 93 cases (5.4%) diagnosed as atypical squamous cells of undetermined significance were positive for HPV67 in cervical-vaginal self-collected samples [28]. These estimates suggest that the rate of HPV67 infection in the general population might be higher than previously thought.

Finally, whether rarely detected but potentially carcinogenic HPVs, such as HPV67, should be targeted in cervical screening tests remains a matter of debate because inclusion of these types in screening tests would decrease specificity and positive predictive value with little or no increase in sensitivity or negative predictive value for detection of ICC or precancer [3, 6]. It should also be examined whether HPV vaccines have cross-protective effects on HPV67 infection given its close phylogenetic relationship with HPV16, 31, 33, 52 and 58, which are all targeted by the 9-valent HPV vaccine. In this regard, neutralization tests using HPV67 pseudovirions will be useful in assessing such cross-protection conferred by the HPV vaccines.

## Conclusion

The current study reported that lineage A variants are dominant among HPV67 infections in Japan and for the first time assessed the transcriptional activity of HPV67 LCR in cervical cancer cells. Given the scarcity of HPV67 complete genome sequences so far, the HPV67 genome sequences that were newly determined in this study will contribute to further functional and evolutionary studies on HPV67 genetic variability.

## Supplementary Information

**Additional file 1**. Multiple sequence alignment of available HPV67 whole genomes.

## Data Availability

The HPV67 whole-genome sequences determined in this study were submitted to the DNA Data Bank of Japan under the accession numbers LC626346 to LC626354 and LC664138 to LC664140.

## Abbreviations

HPV: Human papillomavirus
ICC: Invasive cervical cancer
CIN: Cervical intraepithelial neoplasia
LCR: Long control region
SCC: Squamous cell carcinoma
